# Development and Greenness Evaluation of Spectrofluorometric Methods for Flibanserin Determination in Dosage Form and Human Urine Samples

**DOI:** 10.3390/molecules25214932

**Published:** 2020-10-25

**Authors:** Rasha Ahmed, Inas Abdallah

**Affiliations:** 1 Department of Pharmaceutical Chemistry, Faculty of Pharmacy, Misr International University, Cairo 11341, Egypt; rasha_ahmed@miuegypt.edu.eg; 2 Department of Analytical Chemistry, Faculty of Pharmacy, University of Sadat City, Sadat City 32897, Egypt

**Keywords:** flibanserin, spectrofluorimetry, NEMI, GAPI, urine discipline

## Abstract

Green, economic and sensitive two spectrofluorometric methods were developed for the quantitation of flibanserin (FB) in different matrices, which are based on FB native fluorescence properties. The first technique depends on measuring the relative fluorescence intensity of FB directly at emission and excitation wavelengths(λ_em_/λ_ex_) (371 nm/247 nm), while the second technique is a first derivative (D^1^) spectrofluorometric method, which depends on measuring the peak amplitudes at 351 nm. Linear regressions were observed in the range of 0.1–1.5 μg/mL for both methods. Moreover, both methods were efficiently extended to analyze FB in human urine, indicating the ultra-sensitivity of the methods, and linear regression was found within a range 0.05–0.7 μg/mL for both methods. Excellent selectivity of the proposed methods and good recoveries were obtained upon the analysis of FB in pharmaceutical dosage form and human urine samples without interference from matrix components with acceptable ranges, from 98.86 to 101.46% and from 98.08 to 102.37%, respectively. Greenness of the developed methods was assessed using the national environmental method index (NEMI) and Analytical Eco-scale and Green Analytical Procedure Index (GAPI). The three approaches confirmed that the developed methods are green, safe and environment-friendly.

## 1. Introduction

**Figure 1:**
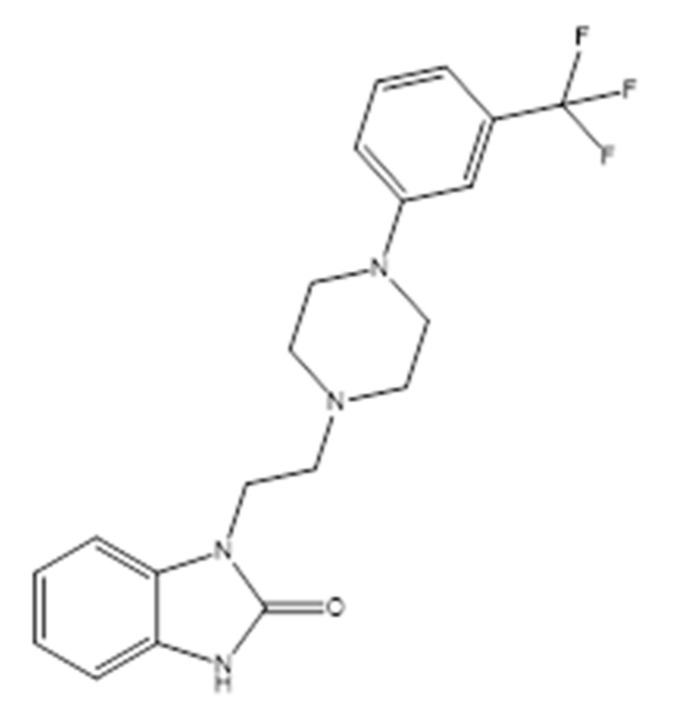
Chemical structure of flibanserin.

Flibanserin (FB) (Figure 1) is a benzimidazole derivative that is chemically known as 1-(2-4-(3-trifluoromethyl-phenyl) piperazin-1-yl)ethyl)benzimidazol-(1H)-2-one [1]. FB improves the sexual desire for premenopausal women suffering from hyposexual disorder by its multifunctional behavior as a serotonin agonist at the 5-HT_1A_ receptors and/or antagonist at the 5-HT_2A_ receptors [2,3]. This behavior causes a reduction in the serotonin level in the brain, as well as the elevation of norepinephrine neurotransmitters, which results in the improvement of sexual functioning in premenopausal women [4,5,6]. Thus, it was approved by the FDA in 2015 for the management of patients with hyposexual desire disorder (HSDD) [7]. The FDA approved a dose of 100-mg FB/day, which is rapidly absorbed after oral administration and excreted as unchanged drug in the urine [8].

Several analytical methods in the literature reported FB determination either in commercial supplements as adulterants or in different biological matrices. These methods were mostly chromatographic methods with different detection techniques [9,10,11]. FB stability was studied with a spectrofluorometric stability, indicating method [12]. Since FB is a recent approved drug, a few analytical methods in the literature study its determination in dosage form or biological samples.

Most of the bioanalytical methods that quantify drugs in biological matrices are chromatographic techniques, which require a long analysis time, high-grade expensive solvents and sophisticated instrumentation [13,14,15]. These drawbacks can be resolved by the use of the sensitive, selective and fast spectrofluorometric technique [16,17,18]. Following oral administration of a single 100-mg dose of flibanserin, the the maximum concentration in plasma (C_max_) was found to be 336 ng/mL. Additionally, the mean terminal half-life of flibanserin after oral administration was approximately 10 h, with 44% excreted in urine and 51% recovered from feces [19]. Due to the high percentage of unchanged excreted FB in the urine, the proposed spectrofluorometric method limit of quantitation will be sufficient for a real sample analysis.

Another important objective was the “greenness evaluation” of the developed spectrofluorometric methods was applied to check these analytical methodology effects on the environment and workers’ health [20].

The aim of this work was to develop green, selective and simple spectrofluorometric methods. These methods can be used in quality control laboratories for the determination of FB in dosage form and used as a bioanalytical method to quantify FB, which is excreted unchanged in urine samples.

## 2. Results and Discussion

Flibanserin was found to exhibit an intense native fluorescence behavior at emission wavelength (λ_em_) = 371nm and excitation wavelength (λ_ex_) = 247 nm. To the best of our knowledge, there is no direct spectrofluorometric method that reported FB quantification in a pharmaceutical dosage form and biological samples. Hence, our work’s objective was to develop two spectrofluorometric methods for the determination of FB in both dosage form and human urine samples. These spectrofluorometric methods depend on the direct method (Method I) and first derivative technique (Method II) without any preliminary separation.

**Figure 2:**
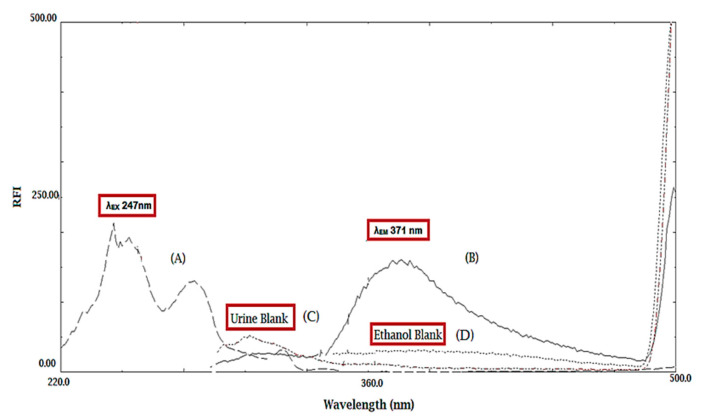
(**A**) Flibanserin (FB) excitation spectrum, (**B**) FB emission spectrum, (**C**) emission spectrum of urine as the blank and (**D**) emission spectrum of ethanol as the blank.

**Figure 3:**
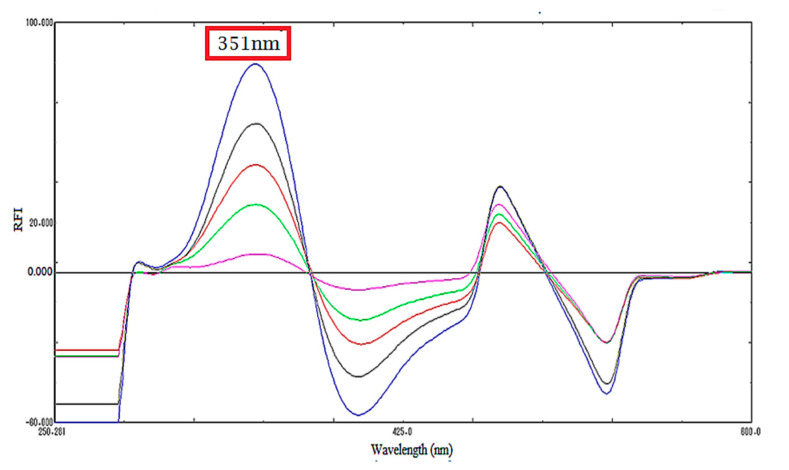
First derivative fluorescence spectra of FB.

Although a third derivative spectrofluorometric method was published before by our research group as a FB stability indicating method [12], in this current work, there was no need for a third derivative transformation of the emission spectra due to the absence of overlapping spectra. Therefore, method I was applied successfully for FB determination in ethanolic solution and human urine, as illustrated in (Figure 2). In addition, the first derivative technique was developed, and the peak amplitudes were measured at 351 nm (method II), as shown in (Figure 3).

During experimental conditions optimizations, the effect of the diluting solvent on FB fluorescence behavior was studied, using different protic and aprotic solvents. Both the relative fluorescence intensity (RFI) and positions of the excitation and emission wavelengths were monitored to study the effect of the diluting solvent. Upon using protic solvents such as ethanol, methanol and water, a solute-solvent interaction takes place due to hydrogen bonding with a variation in the RFI values. When looking at the FB chemical structure, you will find hydrogen-acceptor atoms (1O, 3 F and 4N) that lead to FB-solvent interaction behaviors. It was found that ethanol has the highest RFI value compared to other protic solvents due to the strong hydrogen bonding that is formed between FB and ethanol. When an aprotic solvent was used, such as acetonitrile, the RFI value was very low due to low interaction behaviors. The results are illustrated in Table 1. No change in the position of excitation and emission wavelengths was observed with different diluting solvents. Ethanol was selected as the optimum solvent for the determination of FB spectrofluorimetrically due to the resulting RFI values compared to other solvents.

### 2.1. Method Validation of Spectrofluorometric Methods in Ethanolic Solution

The validation of the developed methods was carried out according to the International Conference on Harmonization (ICH) guidelines [21]. The range, linearity, limit of quantitation (LOQ), limit of detection (LOD), precision and accuracy were investigated.

#### 2.1.1. Linearity

Under the experimental conditions (under Section 3.4), a linear relationship was established by plotting the RFI and peak amplitude (at λ_em_ = 371 nm for method I and λ_em_ = 351 nm for method II) against the corresponding FB concentration. Good linearity for both methods was observed, with correlation coefficients 0.9994 and 0.9991 for methods I and II, respectively. The regression data is illustrated in Table 2 and can be represented by the following equations:

*Y*_1_ = 244.27*x* + 37.41 (Method I at λ_em_ = 371 nm) (1)

*Y*_2_ = 53.99*x* + 1.189 (Method II at λ_em_ = 351 nm) (2)

where *Y*_1_ is the relative fluorescence intensity for method I, *Y*_2_ is the peak amplitude of the first derivative for method II and *x* is the FB concentration in µg/mL.

#### 2.1.2. LOD and LOQ

Estimations of the limit of detection (LOD) and limit of quantitation (LOQ) were calculated by the following equations: LOQ = 10 σ/S and LOD = 3.3 σ/S, where σ represents the standard deviation of the intercept, while S is the slope of the calibration curve. The values of the LOD were found as 0.029 and 0.031 µg/mL, while the LOQ were found as 0.087 and 0.095 µg/mL for FB in method I and method II, respectively.

#### 2.1.3. Accuracy and Precision

Three different concentrations of FB (0.2, 0.5 and 1 μg/mL) were prepared to assess the accuracy of the method by applying the analytical procedures for methods I and II within their linear range. The accuracy was evaluated by calculating both the recovery percentage and standard deviation (SD). The obtained results for the two methods were close to 100%, indicating good accuracy of the proposed methods, as shown in Table 2.

The precision of the proposed methods was evaluated by assessment of the intra-day and inter-day precisions. The intra-day precision was assessed within the same day three different times by determining three different concentrations of the standard FB (0.2, 0.5 and 1 μg/mL) within the linearity range. The inter-day precision was estimated by measuring three replicates of three different concentrations of FB on three different days. In both the intra-day and inter-day precisions of the two methods (I and II), the estimated values of the relative standard deviation (RSD) were lower than 2%, which significantly implies that the presented methods are highly reproducible and precise, as illustrated in (Table 2).

### 2.2. Method Validation of Spectrofluorometric Methods in Human Urine

The proposed spectrofluorometric methods (methods I and II) were applied on the determination of FB in human urine samples. Method validation has to be carried out first in a human urine matrix before the sample analysis, according to the 2019 ICH M10 guidelines [22]. Calibration standards and quality control samples (QCs) were prepared in the same matrix (urine) as the samples to be analyzed. The calibration range was constructed with a range of 0.05–0.7 µg/mL, and the following regression equations of FB in the human urine matrix were calculated for both methods as follows:

*Y*_3_ = 392.71*x* + 108.50 (Method I at λ_em_ = 371 nm) (3)

*Y*_4_ = 61.161*x* + 2.5698 (Method II at λ_em_ = 351 nm) (4)

where *Y*_3_ is relative fluorescence intensity for method I, *Y*_4_ is the peak amplitude of the first derivative for method II and *x* is the FB concentration in µg/mL.

An evaluation of the accuracy and precision for both methods was assessed by analyzing five replicates of QC samples prepared in human urine, with the four concentration levels denoted as lower limit of quantitation (LLOQ), quality control low (QCL), quality control medium (QCM) and quality control high (QCH). The linear regression equations of FB in human urine were used to estimate the drug concentration in each QC sample. No interference was found from the endogenous components of drug-free human urine. The mean recovery percentage values of the QC samples ranged from 99.76 to 102.19% for method I, while ranging from 98.18 to 102.37% for method II, indicating the applicability of the proposed methods for the determination of FB in human urine without interference from urine components. All validation parameters are demonstrated in Table 3.

### 2.3. Applications of the Proposed Spectrofluorometric Methods

#### 2.3.1. Analysis of the Pharmaceutical Dosage Form

The proposed methods were successfully applied for FB determination in commercial dosage form. The obtained percentage recoveries and standard deviations were found as follows: 101.73 ± 0.47 and 99.75 ± 0.28 for methods I and II, respectively. This indicates the accurate determination of FB in dosage form by application of the proposed methods. In addition, the reliability of the proposed methods (I and II) was confirmed by applying the standard addition technique by adding known different concentrations of standard FB to the exact concentration of the pharmaceutical dosage form. The obtained percentage recoveries were found to be acceptable for both methods, as shown in Table 4, indicating no interference from the encountered excipients.

The results of both methods (I and II) were compared to an in-house HPLC method; the values obtained from the proposed methods were compared according to the *t*- and F-tests at a 95% confidence level. No significant difference was found between the proposed methods and the HPLC method, as shown in Table 5.

#### 2.3.2. Analysis in Real Human Urine Samples

Urine samples were collected from five healthy females at different time intervals and prepared as mentioned under Section 3.4.3. The samples were analyzed to determine the concentration of excreted unchanged FB after a single dose of an oral administration of Veroxeserin^®^ (containing 100-mg FB per tablet). The unchanged FB concentration levels were quantified by both methods. The samples ranged in concentrations of 0.064–0.154 μg/mL (Method I), as shown in Table S1 (Supplementary Materials) and 0.074–0.146 μg/mL (Method II), as shown in Table S2 of the unchanged excreted FB. The percentage cumulative of the FB dose excreted was calculated from the regression equation of method I and was found to be 43%, as shown in Table S3, and 41.64% for method II, as shown in Table S4. These percentage cumulative dose-excreted values agreed with the pharmacokinetic excretion data of flibanserin, where 44% of the single oral dose is recovered in the urine [19]. The selectivity and specificity of the method was assured by comparing the mean concentration values obtained from the sample analysis using the proposed methods and an in-house validated HPLC method, as shown in Table S5 and Figure S1. The results of this application prove the applicability of both the spectrofluorometric methods for the determination of FB in human urine samples with no matrix component interference.

### 2.4. Greenness Evaluation of the Proposed Spectrofluorometric Methods

It is very important nowadays to evaluate the greenness of any analytical method and confirm to what extent the method is green. Herein, we applied three approaches to assess the method greenness. The first approach was the national environmental method index (NEMI) [23]. The NEMI is a qualitative tool that illustrates the greenness of any analytical method through a pictogram that is divided into four quadrants and describes (i) PBT: reagents that are not persistent, bioaccumulative or toxic; (ii) hazardous: the reagents are not on the hazardous list, (iii) corrosive: the pH is between 2 and 12 and (iv) waste: the overall waste generated is less than 50 g or mL.

**Figure 4:**
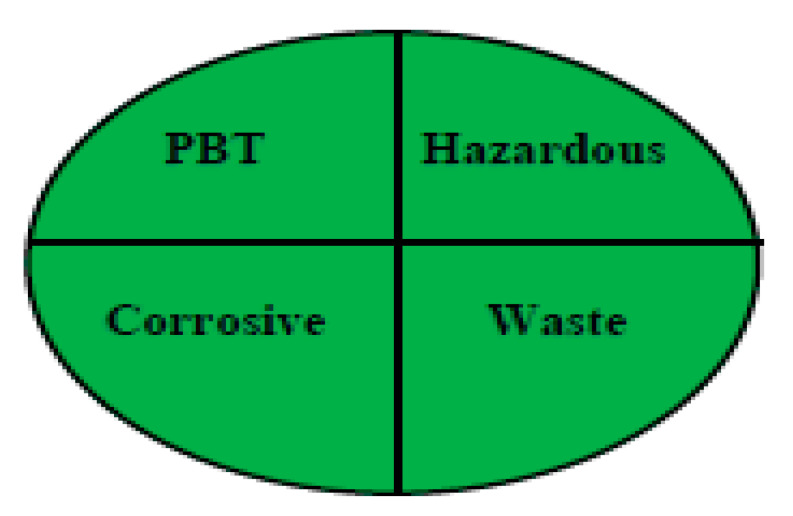
Evaluation of the proposed methods greenness by the national environmental method index (NEMI) pictogram method. PBT: reagents that are not persistent, bioaccumulative or toxic.

The analytical method must satisfy all the above criteria so that all the quadrants are shaded green. As shown in Figure 4, the proposed methods satisfied the criteria of the NEMI approach.

As mentioned before, the NEMI approach is a qualitative approach, which does not take into account the amount of the reagents used, hazardous waste, occupational hazards and the energy consumed. Therefore, a quantitative tool was developed by Van-Aken et al. [24] that is called the analytical eco-scale. It depends on penalty point calculations, which rank the analytical procedure greenness according to the score; methods with a score above 75 are considered excellent green methods, methods with score above 50 are acceptable green methods and methods with a score below 50 are methods with an inadequate greenness profile. Table 6 summarized the penalty points obtained for the proposed methods. Both methods I and II gave a score of 88. This score value confirmed that both spectrofluorometric methods are green methods and environment-friendly.

**Figure 5:**
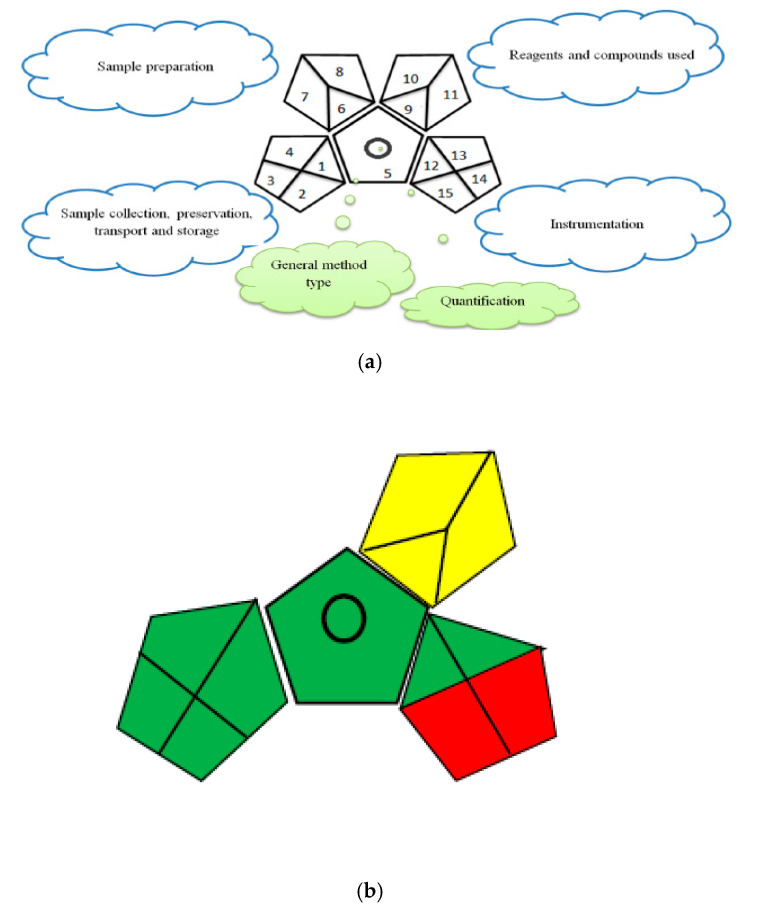
The green analytical procedure index (GAPI) approach for greenness assessment (**a**), with description [25], and (**b**) of the developed spectrofluorometric methods.

The green analytical procedure index (GAPI) was introduced by Plotka-Wasylka [25] as a green tool that combines the advantages of both the NEMI and eco-scale tools and puts into consideration more analytical procedure details to evaluate the method green character. The GAPI evaluates 15 parameters of any analytical procedure, starting with the sample preparation, reagents and solvents, instrumentation, waste and waste treatment. All of these parameters are represented in a colored pictogram depending on their environmental impact, which is either green, yellow or red. The proposed spectrofluorometric methods were applied in the in-line sample collection; the samples did not require preservation, transport or storage. The methods did not require a sample extraction step, which means they were considered as a direct analytical procedure. According to the solvent selection guide [26], green solvent (ethanol) was used in the analytical procedures. All the sections in the pentagrams are colored green, except section 9 was yellow, as the volume of the solvent used in the analytical procedure was 10–100 mL. Section 10 was also yellow-colored, as the ethanol National Fire Protection Association (NFPA) health hazard rating was 2. In addition, section 11 was yellow in color, as the flammability score of ethanol was 3. As the waste volume was >10 mL and no waste treatment was applied, both sections 14 and 15 were colored red. The results of the GAPI assessment are shown in Figure 5 and Table 7.

The greenness assessment of the proposed spectrofluorometric methods by the three previously mentioned approaches proves that the proposed methods are of excellent green practice.

## 3. Experimental

### 3.1. Reagents and Materials

Hikma Pharmaceutical Industries (Cairo, Egypt) kindly supplied the study with flibanserin of purity 99.98% and Veroxeserin^®^ tablets (Batch 001) (each tablet contained 100-mg FB). All solvents used throughout the study (acetonitrile, methanol and ethanol) were of HPLC grade and obtained from Sigma Aldrich (St. Louis, MO, USA).

### 3.2. Instrumentation

Shimadzu spectrofluorometer (model: RF 5301 PC, Japan) was used for fluorescence measurements. The instrument was equipped with 150-watt Xenon lamp, and a 1-cm quartz cell was used. Slit widths for excitation and emission monochromators were set at 5 nm.

### 3.3. Preparation of a Standard Solution of FB

A stock standard ethanolic solution of FB (100 µg/mL) was prepared by dissolving 10 mg of FB in a 100-mL volumetric flask and completed to the mark with ethanol. Accurate volumes from FB stock standard solution were diluted for the preparation of working standard solutions of concentrations ranging from 5–70 µg/mL.

### 3.4. Procedure

#### 3.4.1. Calibration Curves

Method I: Aliquots of FB working standard solutions were transferred to 10-mL volumetric flasks and completed to the mark with ethanol to reach final concentration ranges of 0.1–1.5 μg/mL. Fluorescence intensities were measured at λ_em_/λ_ex_ 371 nm/247 nm, and the calibration curve was constructed between fluorescence intensity against the corresponding concentrations; then, the regression equation was computed.

Method II: Standard solutions of FB with final concentration ranges of 0.1–1.5 μg/mL were prepared by transferring aliquots of FB working standard solutions to a 10-mL calibrated volumetric flask and diluted to the mark to reach the final concentrations. Peak amplitude of the first derivative emission spectra (D^1^) was measured at 351 nm. Regression equation was computed from a constructed calibration curve between the peak amplitudes and concentration.

#### 3.4.2. Analysis of FB in Pharmaceutical Dosage Form

A weight of finely powdered ten Veroxeserin^®^ tablets equivalent to 100 mg of FB was transferred to a 100-mL volumetric flask. The content was diluted with 50-mL ethanol and sonicated for 30 min; then, the volume was completed with the same solvent to reach a final concentration of 100 µg/mL. The solution was filtered, and an accurate aliquot of FB filtrate (0.05 mL) was transferred into a set of 10-mL calibrated volumetric flasks, then completed with ethanol to give a concentration of 0.5 µg/mL. The general procedure was followed for either method I or method II to check the claimed concentration of FB in tablets using regression equations for each method separately.

#### 3.4.3. Assay of FB in Human Urine and the Application to Real Samples

A volume of 900 μL of free human urine was transferred into 100-mL calibrated volumetric flasks, then 100 μL of different concentrations of working standard solutions of FB (5–70 μg/mL) were added. Each solution was diluted with ethanol and vortexed for 5 min, then completed to the mark to reach a final concentration range of 0.05–0.7 μg/mL. The general procedures of methods I and II were followed, and the regression equations were computed as described previously.

Urine samples were collected from five healthy, nonalcoholic and nonsmoking female volunteers. The urine samples were collected from the volunteers after administration of a Veroxeserin^®^ tablet. The samples were collected using a polypropylene centrifuge tube at 0–2, 2–4, 4–6, 6–8, 8–10 and 10–12 h past the drug administration. Finally, 1 mL of each urine sample was filtered with a 0.45-μm syringe filter and transferred to a 100-mL volumetric flask. The content was diluted with ethanol to the mark; then, the samples were treated as previously described under the procedures of method I and II. Then, the excreted unchanged concentration was calculated.

## 4. Conclusions

This work described two green, sensitive, selective and cost-effective spectrofluorometric methods that were applied for FB determination in a dosage form. In addition, owing to their high sensitivity, this work extended to analyze real human urine samples with good percentage recoveries and with no need for pretreatment steps. No interference was found from the tablet excipients or urine components. Both methods were assessed for their greenness, and they were found to be green and environment-friendly.

Therefore, the proposed methods can be successfully applied for the determination of FB in quality control labs and in the clinical analysis of FB through the establishment of a urinary excretion pattern.

## Figures and Tables

**Table 1:** Effect of the solvents on the relative fluorescence intensity of flibanserin (FB).

Type of Solvent	RFI *
Water	80
Methanol	156
Ethanol	200
Acetonitrile	89

* RFI: relative fluorescence intensity.

**Table 2:** Analytical parameters of the proposed spectrofluorometric methods in the ethanolic solution.

Parameter	Method I	Method II
λ_ex_	247	247
λ_em_	371	351
Linearity:		
Regression equation	*Y*_1_ = 244.27*x* + 37.41	*Y*_2_ = 53.99*x* + 1.189
Range (μg/mL)	0.1–1.5	0.1–1.5
Correlation coefficient (r)	0.9994	0.9991
Slope (b)	244.27	53.99
Intercept (a)	37.41	1.189
SD of slope (S_b_)	2.528	0.616
SD of intercept (S_a_)	2.114	0.515
LOD (μg/mL)	0.029	0.031
LOQ (μg/mL)	0.087	0.095
Precision:		
Repeatability (Intraday) (%RSD) *		
QCL (0.2 μg/mL)	0.41%	1.39%
QCM (0.5 μg/mL)	0.50%	0.95%
QCH (1 μg/mL)	0.20%	1.40%
Intermediate precision (Inter-day) (%RSD) *		
QCL (0.2 μg/mL)	0.38%	0.19%
QCM (0.5 μg/mL)	0.18%	0.38%
QCH (1 μg/mL)	0.43%	0.31%
Accuracy: (Mean ± SD) **		
QCL (0.2 μg/mL)	101.09 ± 1.13	101.23 ± 0.89
QCM (0.5 μg/mL)	99.08 ± 0.55	99.03 ± 0.91
QCH (1 μg/mL)	101.37 ± 0.60	100.89 ± 1.41

* RSD: relative standard deviation. ** Expressed mean of three replicates. LOD: limit of detection and LOQ: limit of quantitation. QCL: Quality control low. QCM: Quality control medium. QCH: Quality control high.

**Table 3:** Analytical parameters of the proposed spectrofluorometric methods in human urine.

Parameter	Method I	Method II
λ_ex_	247	247
λ_em_	371	351
Linearity:		
Regression equation	*Y*_3_ = 392.71*x* + 108.50	*Y*_4_ = 61.161*x* + 2.5698
Range (μg/mL)	0.05**–**0.7	0.05**–**0.7
Correlation coefficient (r)	0.9991	0.9990
Slope	392.71	61.161
Intercept	108.50	2.5698
QC samples:		
Repeatability (Intra-day)	Accuracy * ± CV% **	
LLOQ (0.05 μg/mL)	100.41 ± 0.67	100.66 ± 1.62
QCL (0.1 μg/mL)	99.76 ± 1.38	102.19 ± 0.42
QCM (0.3 μg/mL)	102.04 ± 0.58	102.03 ± 0.56
QCH (0.6 μg/mL)	100.56 ± 0.44	98.08 ± 0.98
Intermediate precision (Inter-day)	Accuracy * ± CV% **	
LLOQ (0.05 μg/mL)	100.57 ± 0.84	100.93 ± 1.59
QCL (0.1 μg/mL)	100.10 ± 1.03	102.84 ± 1.28
QCM (0.3 μg/mL)	102.28 ± 0.57	102.37 ± 0.74
QCH (0.6 μg/mL)	100.44 ± 0.42	98.18 ± 0.68

* Average of five replicates. ** Coefficient of variation. LLOQ: Lower limit of quantitation. QCL: Quality control low. QCM: Quality control medium. QCH: Quality control high.

**Table 4:** Application of the proposed methods (I and II) for the determination of FB in a pharmaceutical dosage form and application of the standard addition technique.

Method	Labeled Content *	Found (Mean ± SD)	Standard Addition Technique	
			Pure Added (μg/mL)	%Recovery ** (Mean ± SD)
Method I	100 mg	99.19 mg ± 0.65	0.3	98.86 ± 1.07
			0.5	101.46 ± 0.95
			0.8	100.86 ± 0.26
Method II		98.98 mg ± 0.90	0.3	100.47 ± 1.36
			0.5	99.21 ± 1.67
			0.8	100.85 ± 0.58

* Pharmaceutical dosage form: Veroxeserin^®^ tablets (Batch number 001) labeled content = 100 mg/tablet. ** Average of three determinations.

**Table 5:** Statistical comparison of the results obtained by the proposed method and in-house HPLC method for determination of FB in Veroxeserin^®^ tablets.

Parameter	Method I	Method II	In-House HPLC Method *
Mean	99.19	98.98	98.36
SD	0.65	0.90	0.15
Variance	0.53	0.81	0.38
N	5	5	5
Student’s *t*-test **	2.26	1.43	-
	(2.306)	(2.30)	
F-test **	3.63	5.54	-
	(6.388)	(6.388)	

* An Eclipse XDB C18 column (150 *×* 4.6 mm, 5 µm) using a mobile phase composition of methanol: 0.05-M ammonium acetate buffer (pH 4.0) (90:10, *v/v*). The flow rate is 1 mL/min, and wavelength of the detection is 237 nm. ** The values in the parenthesis are the corresponding theoretical values of the *t*- and F-tests at *p* = 0.05.

**Table 6:** The penalty points of the proposed methods according to the analytical eco-scale.

Reagents/Instruments	No of Pictograms	Word Sign	Penalty Points (Methods I and II)
Reagents			
Ethanol	2	Danger	
Instrument Spectrofluorometer			0
Occupational hazard			0
Waste			8
Total penalty points			∑ 12
Analytical eco-scale total score			100 − 12 = 88

**Table 7:** Green analytical procedure index parameters for the developed methods.

Category	Methods I and II
Sample preparation	
1- Collection	In-line
2- Preservation	None
3- Transport	None
4- Storage	None
5- Type of the method (direct/indirect)	Direct (No sample preparation)
6- Scale of extraction	None
7- Solvents/ Reagents	Solvent-free extraction method
8- Additional treatments	None
Reagents and solvents	
9- Amounts	10–100 mL
10- Health hazard	Ethanol: Slightly toxic, slightly irritant, NFPA health hazard rating = 2
11- Safety hazard	Ethanol: instability score = 0, Flammability score = 3, No special hazard
Instrumentation	
12- Energy	≤0.1 kWh/sample
13- Occupational hazard	Hermetic sealing of the analytical process
14- Waste	>10 mL
15- Waste treatment	No treatment
Additional mark: Quantification Circle in the middle of GAPI: procedure for qualification and quantification	

NFPA: National Fire Protection Association. GAPI: the green analytical procedure index.

## Supplementary Materials

The following are available online: Table S1: The concentration of FB excreted in urine samples in (µg/mL) as determined by Method I. Table S2: The concentration of FB excreted in urine samples in (µg/mL) as determined by Method II. Table S3: Cumulative percentage amount of FB (mg) excreted in urine of female volunteers following oral administration of 100 mg tablet by Method I. Table S4: Cumulative percentage amount of FB (mg) excreted in urine of female volunteers following oral administration of 100 mg tablet by Method II. Table S5: Comparison between Method I and I for determination of the average concentration of FB excreted in urine samples in (µg/mL) and HPLC method. Figure S1: Representative HPLC Chromatogram (a) Blank human urine and (b) urine sample obtained after 10 h of FB single dose (100 mg FB/ tablet).
