# Supplementary material for: Development and Greenness Evaluation of Spectrofluorometric Methods for Flibanserin Determination in Dosage Form and Human Urine Samples
Source: Molecules. 2020 Oct 25;25(21):4932. doi: 10.3390/molecules25214932 (PMC7663165; doi:10.3390/molecules25214932)
Supplement: Supplementary file 1 [file molecules-25-04932-s001.pdf]

SUPPORTING INFORMATION

Development and Greenness Evaluation of Spectrofluorometric methods for Flibanserin Determination in Dosage Form and Human Urine Samples

Rasha Ahmed[1], Inas Abdallah[2*]

[1]Department of Pharmaceutical Chemistry, Faculty of Pharmacy, Misr International University, Cairo, Egypt.
[2]Department of Analytical Chemistry, Faculty of Pharmacy, University of Sadat City, Sadat City, Egypt.

# Corresponding author

Inas A. Abdallah

Department of Analytical Chemistry, Faculty of Pharmacy, University of Sadat City, Sadat City, Egypt

E-mail: inas.abdallah@fop.usc.edu.eg

Table S1: The concentration of FB excreted in urine samples in (µg/mL) as determined by Method I

Collection interval (hours)						
**Volunteers**	**0 - 2**	**2 - 4**	**4 - 6**	**6 - 8**	**8 - 10**	**10 - 12**
**1**	ND	0.08	0.09	0.095	0.11	0.15
**2**	ND	0.06	0.10	0.11	0.12	0.14
**3**	ND	0.05	0.12	0.13	0.095	0.16
**4**	ND	0.06	0.11	0.09	0.13	0.15
**5**	ND	0.07	0.09	0.095	0.10	0.17
**Mean**	-----	**0.064**	**0.102**	**0.104**	**0.111**	**0.154**
**± SD**	------	**0.011**	**0.013**	**0.016**	**0.014**	**0.011**

*ND: FB is not detected in urine samples.

Table S2: The concentration of FB excreted in urine samples in (µg/mL) as determined by Method II

	Collection interval (hours)					
**Volunteers**	**0 - 2**	**2 - 4**	**4 - 6**	**6 - 8**	**8 -10**	**10 - 12**
**1**	ND	0.09	0.09	0.095	0.12	0.15
**2**	ND	0.05	0.09	0.1	0.11	0.16
**3**	ND	0.09	0.10	0.11	0.12	0.14
**4**	ND	0.08	0.095	0.095	0.11	0.15
**5**	ND	0.06	0.08	0.085	0.095	0.13
**Mean**	**-----**	**0.074**	**0.091**	**0.097**	**0.111**	**0.146**
**± SD**	**------**	**0.018**	**0.007**	**0.009**	**0.010**	**0.011**

*ND: FB is not detected in urine samples.

Table S3: Cumulative percentage amount of FB (mg) excreted in urine of female volunteers following oral administration of 100 mg tablet by Method I

Collection intervals (hours)	Concentration (μg/mL)	Volume (mL)	Amount (μg)	Amount (mg)	Cumulative amount (mg)
**0 - 2**	ND	800	-----	------	------
**2 - 4**	0.064	750	4800	4.8	4.8
**4 - 6**	0.102	800	8160	8.16	12.96
**6 - 8**	0.104	850	8840	8.84	21.80
**8 -10**	0.111	800	8880	8.88	30.68
**10 - 12**	0.154	800	12320	12.32	43

$$\boldsymbol{Percentage\ cumulative\ dose\ excreted = \frac{Cumulative\ amount\ excreted\ (mg)}{Total\ dose\ given\ (mg)}\ x\ 100}$$

$$\%\ cumulative\ dose\ excreted = \frac{43}{100}\ x\ 100 = 43\ \%$$

Table S4: Cumulative percentage amount of FB (mg) excreted in urine of female volunteers following oral administration of 100 mg tablet by Method II

**Collection intervals (hours)**	**Concentration (µg/mL)**	**Volume (mL)**	**Amount (µg)**	**Amount (mg)**	**Cumulative amount (mg)**
**0 - 2**	ND	800	-----	------	------
**2 - 4**	0.074	750	5550	5.55	5.55
**4 - 6**	0.091	800	7280	7.28	12.83
**6 - 8**	0.097	850	8245	8.25	21.08
**8 - 10**	0.111	800	8880	8.88	29.96
**10 - 12**	0.146	800	11680	11.68	41.64

$$\boldsymbol{Percentage\ cumulative\ dose\ excreted = \frac{Cumulative\ amount\ excreted\ (mg)}{Total\ dose\ given\ (mg)}\ x\ 100}$$

$$\%\ cumulative\ dose\ excreted = \frac{41.64}{100}\ x\ 100 = 41.64\ \%$$

Table S5: Comparison between Method I and I for determination of the average concentration of FB excreted in urine samples in (µg/mL) and HPLC method.

Collection intervals (hours)	Method I	Method II	HPLC method*
	Mean ± SD		
**0 - 2**	ND	ND	
**2 - 4**	0.064 ± 0.011	0.074 ± 0.018	0.069 ± 0.007
**4 - 6**	0.102 ± 0.013	0.091 ± 0.007	0.097 ±0.008
**6 - 8**	0.104 ± 0.016	0.097 ± 0.009	0.101 ± 0.005
**8 - 10**	0.111 ± 0.014	0.111 ± 0.010	0.113 ± 0.005
**10 - 12**	0.154 ± 0.011	0.146 ± 0.011	0.15 ± 0.006

* An Eclipse XDB C18 column (150 x 4.6 mm, 5µm) using a mobile phase composition of (methanol: 0.05 M ammonium acetate buffer (pH 4.0)) (90:10, v/v). The flow rate is 1mL/min and wavelength of detection is 237nm.

a

b

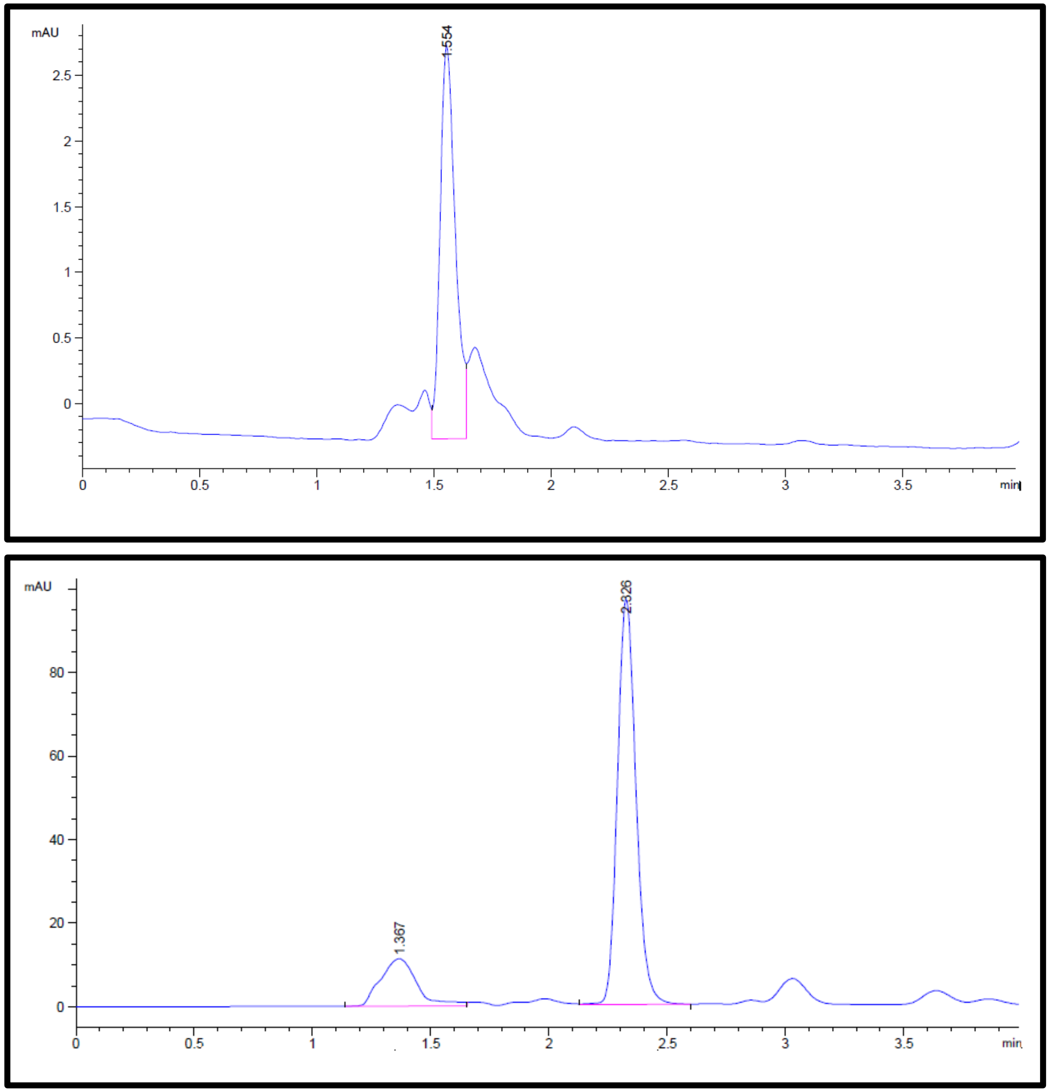

Figure S1- Representative HPLC Chromatogram (a) Blank human urine and (b) urine sample obtained after 10 hours of FB single dose (100 mg FB/ tablet).
